# Promoting language development in physically disabled adults through sports: The content and language integrated learning method

**DOI:** 10.3389/fpsyg.2022.969877

**Published:** 2022-10-21

**Authors:** Hasan Bekirogulları, Nazım Burgul, Emete Yagcı

**Affiliations:** Department of Physical Education, Near East University, Nicosia, Cyprus

**Keywords:** psychology of education, motivation, language learning, affective factors, content and language integrated learning (CLIL)

## Abstract

This study reports on the outcomes of a qualitative study which explores the perceptions of ten wheelchair basketball players of the implementation of a content and language integrated learning (CLIL) program. The participants were all members of the official wheelchair basketball team of the Sports Federation for the Disabled in North Cyprus. They were all young adults (18–35 years old) and their level of English language proficiency ranged from elementary to beginner. After a 4-month CLIL intervention period, the data were elicited through individual interviews from the participants. A qualitative analysis of the textual data revealed the themes such as improved self-image, high motivation, developed social skills, and better speaking skills in the target language. The results have a few practical implications for English such as foreign language teachers, sports coaches, and local disability committees.

## Introduction

There has been a growing trend in education to teach subjects other than languages by adopting a foreign language as the medium of instruction. Content and language integrated learning (CLIL) has been popular lately and is viewed as an innovative method of teaching languages (Le and Nguyen, 2022). CLIL practices aim not only to improve language skills but also to enhance academic cognitive processes and intercultural understanding (Gabillon and Ailincai, 2013). CLIL programs encompass the integration of a subject with foreign language teaching, mainly English, which entails the cooperation between language teachers and content teachers. CLIL is welcomed worldwide as a popular approach that promotes foreign language learning. Indeed, according to Dalton-Puffer (2007), foreign language learning occurs naturally in the CLIL classroom. During CLIL methodologies, the focus is on meaning rather than form; hence, students are provided with a purpose to use the foreign language (Alexiou, 2015). Students learn a particular subject by being exposed to a foreign language through CLIL programs. The main goal of the integration of CLIL programs is to teach subject area knowledge while improving students’ foreign languages.

Content and language integrated learning makes way for incidental and unplanned language learning in a natural way (Coyle et al., 2010, p. 18). CLIL has the potential to transform the dynamics of learning, making it more motivational and constructivist due to the use of real language (Pérez-Cañado, 2011). There are clashing views on the influence of CLIL on sports. While some argue that students’ intrinsic motivation has a positive effect on English learning (Coral, 2010), some studies indicate that learning a foreign language could be stressful and causes anxiety (Figueras et al., 2011).

Sport has the power to unite people regardless of gender, color, and race. Sport, more than any other discipline, is a vehicle for inclusion even for the disabled individuals. The CLIL classroom also has a potential for inclusion, leveling up, and reducing inequality. In this respect, it is imperative to design studies exploring the effectiveness of teaching sports with the adoption of a foreign language *via* CLIL programs. CLIL programs focus on four competencies, namely, content, cognition, communication, and culture. The major aim of CLIL programs is to develop communicative and cognitive skills in the target language while building knowledge of the subject. Although CLIL is widely used in Europe when teaching arts, biology, chemistry, and social sciences, studies on the adoption of a CLIL approach in physical education and sports classes are not frequently cited. Overall, less CLIL research has been conducted on sports for the disabled individuals. To address this void in the literature, this study is designed with the aim of exploring the physically disabled individuals’ perspectives regarding the effects of CLIL on their morale, motivation, wellbeing, and linguistic development.

### Research questions

To fulfill the research aim, the following questions were posed:

1. In what ways does CLIL intervention affect wheelchair basketball players?
2. To what extent does CLIL intervention benefit wheelchair basketball players in terms of linguistic gains?

## Review of literature

Language and content development is the main focus of CLIL. Students are exposed to a foreign language, while they are dealing with a variety of topics in CLIL classes. In addition, since they are exposed to two languages (one being their mother tongue and the other being a foreign or a second language), at the same time, their thinking skills in these languages develop as well. CLIL classes also enhance linguistic competence (Lasagabaster, 2008; Harrop, 2012) as students engage in meaningful interaction (Dale and Tanner, 2012). Sakellariou and Papadopoulos (2020) acknowledged the benefits of CLIL regarding language and cognitive development. Using the total physical response methods in physical education lessons, CLIL has the potential to improve students’ English language comprehension (Asher, 2003). A great number of vocabulary input foster vocabulary development and knowledge during CLIL instruction (Alexiou and Stathopoulou, 2021). This, in turn, adds to the development of speaking skills (Lasagabaster, 2008).

Research conducted with tertiary students studying at the Faculty of Sports is highly satisfied with the CLIL approach in Barcelona (Figueras et al., 2011). According to Dalton-Puffer (2007), in CLIL classrooms, unlike language classrooms, students use the target language naturally, just like they use their mother tongue naturally in everyday life. Similarly, Christopher et al. (2012) delved into teaching English through sports to higher education students to find that the participants gained confidence in terms of speaking English. The reason for increased speaking skills may be the real-life situations that sports provide and the fact that both language time exposure and oral interaction are increased in such learning environments (Mateu, 2013). When using a variety of vocabulary and grammatical structures, the coach naturally provides necessary linguistic input for the participants. Research indicates favorable results toward CLIL learners (Dalton-Puffer, 2007; Lasagabaster, 2008). More specifically, these studies indicate that CLIL learners acquire the knowledge of the subject as successfully as those who study in their mother tongue. Furthermore, Lasagabaster’s (2011) study found that CLIL learners even outperform students in controlled groups regarding the development of language and cognitive skills.

The main idea behind the use of CLIL is to prepare the participants for a better role in society. Learning a foreign language helps learners to understand the multicultural world, gain self-confidence, and develop linguistic achievement and self-expression. Owing to all these good qualities and life skills that CLIL provides, it has the potential to improve the lives of the disabled individuals in terms of socialization and motivation. Christopher et al. (2012) explored teaching English through sports to higher education students to find that the participants have gained confidence in terms of speaking English.

The interaction between the coach and the players develops social skills. CLIL programs can also contribute to forming strong coach–athlete relationships. Forming effective coach–athlete relationships benefits the team by building motivation and morale. Research shows that since students are engaged in cooperative learning during CLIL, they can develop their communicative competence, social and linguistic skills, as well as interdependence (Martínez, 2011). Another advantage of CLIL is that participants develop a strong sense of achievement as they see their own progress in the target language (Dale and Tanner, 2012).

Harrop’s (2012) study indicated that CLIL programs foster motivation as well as greater intercultural awareness. CLIL instruction makes way for intercultural awareness (Lasagabaster, 2008). Diab et al. (2018) argued that learning a language is inseparable from learning the culture and that the culture awareness development is facilitated by the CLIL method. Intercultural understanding, personal development, cooperation, teamwork, and social interaction are inherent in the culture. As argued by Mateu (2013), sport provides a motor content, and developing such a motor content in the CLIL classroom through a foreign language contributes to international understanding since the foreign language sets the context of the content in a different culture. According to Harrop (2012), even learning a foreign language is part of the intercultural learning process and this intercultural awareness has the potential to change students’ outlook.

## Methods

This study focused on wheelchair basketball players’ perceptions regarding CLIL instruction. Considering the aim of the study to explore the physically disabled young adults’ perspectives regarding the effects of CLIL on their morale, motivation, wellbeing, and linguistic development, a qualitative research method was chosen. As argued by Halcomb and Davidson (2006), qualitative research methods focus on the exploration of perceptions, meanings, beliefs, experiences, and feelings.

### Subjects

The sample was composed of 10 physically disabled wheelchair basketball players. They were all young adults (18–35 years old). Their level of English language proficiency ranged from elementary to beginner. All of them were male.

### Context

The Sports Federation for the Disabled in north Cyprus was established in 1996 with the mission of introducing sports to all disabled citizens of north Cyprus and to equip them with a new outlook on life. There is only one official wheelchair basketball club that is a member of the federation. There is also another club but it is not a member of the federation. Only the official club’s players took part in this study. The official wheelchair basketball club competes with those in Turkey and once they participated in a tournament in Italy as a Turkish team.

Following an ethical approval granted by a higher education institution in north Cyprus, the authorities of the Sports Federation for the Disabled in north Cyprus were contacted and a meeting was arranged with the players and the coach. During the meeting, the aim of the study was clarified and they were briefed about the nature of the intervention. What they needed to do during the training sessions was explained in detail. More specifically, they were told that they would keep going with their usual training sessions with their coach and that the only difference would be that they would be accompanied by an English-speaking assistant coach. It was explained to them that there was no expected harm from the study but they could benefit by learning or practicing English so that they would be able to understand basketball terminology during the tournaments in Europe. In fact, sports teams in north Cyprus cannot participate in European cups due to the embargo, but, especially, teams such as the disabled basketball team can compete if they were a Turkish team. They were also informed that participation was on a voluntary basis and that if they consented, they would be interviewed at the end of a 4-month period.

### Procedure

This study partially applies Mateu’s (2013) model. Learning outcomes were identified as “what students should know,” “be able to do,” and “be aware of at the end of the unit” (Mateu, 2013). More specifically, they should know the basic commands like run, catch, and throw, “be able to do” refers to understanding commands, and “be aware of at the end of the unit” refers to the awareness of communicating in English. The participants were expected to acquire basketball terminology, such as violation, turnover, traveling, time out, three-point line, technical foul, substitute, slam dunk, shot clock, shoot, set shot, referees, rebound position, personal foul, overtime, overhead pass, no-look pass, MVP, lay-up, jump shot, hoop, guarding, game clock, free-throw, foul, exceed, drive, dribble, draft, double-dribbling, chest pass, bounce pass, bounce, block, basket, backboard, and assist. The language structures were identified as *Bounce the ball*, *we won*, *throw a chest pass*, *run forward*, *don’t exceed the time limit*, *Do you have any fouls?*, *I have two fouls*, *we lost possession of the ball*, *the coach gets a technical foul*, *don’t violate the rules*, *let’s play*, and *it’s my mistake*. Language functions were giving commands, asking and answering questions, giving opinions, and explaining. As Coyle et al. (2010) argued, “Developing a repertoire of speech acts which relate to the content, such as describing, evaluating, and drawing conclusions, is essential for tasks to be carried out effectively.”

The training sessions were designed to pay attention to content, communication, cognition, and culture developed by Coyle et al. (2010). In this respect, developing content knowledge, and language skills as well as communication within the content were paid special attention. During the introductory phase, the coach defined the content to be taught to the participants in Turkish and then using Total Physical Response (TPR), the assistant coach first modeled the move while naming the move in English and then asked them to do so while repeating it after him. Thus, team members construct their knowledge by active participation. The whole procedure lasted for 4 months, and the training sessions were held three times a week.

### Data collection

To evaluate the effectiveness of the CLIL experience, semi-structured interviews were conducted with each participant. Creswell (2012) noted that semi-structured interviews permit the participants to describe the details for personal information, and the interviewer has good control over the information received. Open-ended questions, such as “How would you describe this experience?, How did you benefit from this experience?, What challenges have you experienced?,” were asked. The interviews took around 45 min and were recorded, transcribed, and translated into English. For ethical issues, all participants took place in the study with pseudonyms.

### Data analysis

Once the interview data were transcribed, a preliminary content analysis was conducted following the Halcomb and Davidson’s (2006) model. A series of categories and subcategories were identified. The data were coded separately by each researcher and then standardization sessions were conducted following a detailed discussion until an agreement concerning the subcategories was reached. Then, the themes were elicited.

## Findings

The analysis of the data revealed four themes, namely, improved self-image, high motivation, developed social skills, and better speaking skills. The findings were revealed to be discussed by theme:

### Improved self-image

Overall, all participants reported positive perceptions of the CLIL experience in terms of self-image. Ali stated,

I never thought it would be possible for me to be a basketball player or to play in Turkey or abroad. I was tired of everything and I did not have anything to do. I lost my purpose in life and every day was the same. I didn’t even want to get up when I woke up. this was what life was like before I started playing basketball. It was really hard first and then with the help of the coach we got better and better each day and now I am also learning English through Basketball. It is something different… the assistant coach was really fun so he helped us a lot. I told him several times that I can’t talk …he said easy easy. Although I learnt little English at school I couldn’t speak or understand any word other than yes and no but in time I learnt at least some words. To manage something is really good, especially for the likes of me. One feels valuable.

Similarly, Osman acknowledged a better self-image,

I think I’m getting better and better every day at basketball. At first it was awful for me because I have never played any sports when I was a child. Being a basketball player was like a dream to me even now I can’t believe that I turned into a basketball player. Although I am still trying hard I know that if I work hard I can manage something. Learning English was fun and I enjoyed it with the assistant coach. He is a nice person. I did not speak English after I left school and I thought I forgot. I will study English more because I will need it abroad. Having a purpose made me a better person. My parents objected my playing basketball first but now they encourage me to attend the trainings and they come to watch my games. My boring life changed.

Arin responded,

I feel so good about this experience… obviously it’s added to my life …it’s added to me because playing basketball gives me the feeling of achieving something in life because I was like nobody. I am a basketball player now. I didn’t achieve anything and I have never been successful in my life in anything but to be able to play basketball to learn English makes me feel like other normal people. Seeing that I can play basketball and I can speak English makes me believe that I can do the things that I’d like to do in life so this gives me the feeling that anything is possible also for me. I feel stronger.

Kemal also expressed improved self-esteem,

I really liked speaking English after a very long time. Learning English brings something else to our training sessions, a good change. We also enjoyed ourselves and I did not feel shy because the assistant coach helped us a lot. He is a very nice guy, he never got tired and he repeated each word several times until we got it. I like English and especially learning English words for basketball was very useful for me. That I can play basketball and learning English changed my mood and I started to believe in myself.

### High motivation

The second theme that emerged was high motivation. All participants stated that the CLIL experience was very useful and enjoyable. Burak replied,

I enjoyed myself during the training with the assistant coach. He made learning English fun. A different experience from the English class at school, we never got bored.

Sinan commented,

I couldn’t wait for the next training session. We all had fun, we laughed a lot at the same time we learnt a lot of words, English words, while playing basketball.

Ali added,

I wish we had such fun English classes at school because if we had learnt English that way I would have been much better at English now. You know at school everything is very serious, you have exams. Now we learn English without any exams and we learn it in a relaxed platform. That is good because you learn it without memorizing, just naturally. That is enticing.

Mert said,

I think it’s a very interesting idea to use English when teaching basketball because it makes it more enjoyable and fun and interesting. I was looking forward to my next training session. I must also give credit to the assistant coach who is a very nice person.

### Developed social skills

Wheelchair basketball players benefit from basketball instruction since basketball like any other competitive sport is a valuable team sport, which makes way for collaboration and group work activities. Collaboration and cooperation increase the coach–player and player–player interactions. Most participants mentioned the importance of socialization during the interviews. Saner reported,

We are all friends with my team mates. We are like a family now. Our parents made friends too because they all come to watch us during the games. They all met so we are a very big family now. I always feel their support.

Sean stated,

Sometimes they invite us as a team for certain organizations. We meet new people and make friends. We also receive invitations from Turkey. We met other wheelchair basketball players in Turkey.

Ahmet told,

People come to watch the games and meet us afterward, which makes me proud.

As reported by Kemal,

My life is better now because I can meet my friends during the day. I attend my training sessions and I learn English so I have things to do, which are very meaningful to me.

Mert commented,

When you do not have anything to do, every day is the same. For people with disabilities there is not much to do in here. Basketball gave my life back to me. I do not want to sit at home anymore. I can mix with them.

### Better speaking skills

The wheelchair basketball players had positive perceptions of CLIL, and they highlighted that their English language development was enhanced. Most of them reported that such training sessions developed English and gave them the opportunity to practice English during training sessions. Osman stated,

I was not really good at English at school so at first I was not sure about myself. The assistant coach was very patient with me but 1 or 2 weeks later I’ve got used to it. Besides he uses some words very frequently like good man nice shot. I just learnt basic words naturally.

Burak told,

These 4 months were really useful for me to develop my English. We learnt simple English, every day English, and spoken English only. You do not need to know perfect English to communicate with people. Even in Turkish we use a limited number of words.

## Discussion

One of the major results of this study was that the CLIL experience resulted in improvements in the wheelchair basketball players’ feelings about themselves, noticing that they could also achieve something when given an opportunity made them proud. Despite the fact that some of them believed they could never learn English, they could at least understand and perform the given commands after 4 months of exposure to English, which made them hopeful about themselves. This finding went in line with that of Dale and Tanner (2012) that CLIL learners developed a strong sense of achievement when they saw their own progress in the target language.

Another result of the study was that all participants were highly motivated. Motivation plays a great role in the learning process (Subramaniam and Silverman, 2007). The wheelchair basketball players stated during the interviews that motivation was high during their CLIL experience. They acknowledged the role of learning English and the assistant coach in their motivation. This finding went in line with that of Perlman (2015) that different forms of teacher instruction had a substantial influence on students’ motivational responses. This finding corroborated that of other studies (Figueras et al., 2011; Harrop, 2012; Zindler, 2013) that introducing a foreign\second language could have a positive influence on increasing motivation.

Furthermore, the results indicated that through the cooperative and collaborative nature of basketball, social interaction was fostered. This finding went in line with previous studies (Coral and Lleixà, 2016) that emphasized the important role of the language. Through cooperative and collaborative group work, the participants had to engage in meaningful interaction with their peers, which increased language use as well as social participation. The finding that CLIL developed social skills corroborated that of Martínez (2011) that cooperative learning inherent in CLIL made way for the development of social skills.

Finally, it was found that CLIL benefited the participants in terms of linguistic gains. That the participants reported developed linguistic skills could be acknowledged by the fact that CLIL provided real-life environments, making way for language development. This finding supported Massler (2012) that CLIL learners could use the skills to learn a foreign language in real-life situations. Besides, all the participants liked the assistant coach because he provided an enjoyable learning environment for them. They were free to make mistakes and this relaxed and informal environment could have fostered more interaction in the target language. This finding supported Harrop (2012), Lasagabaster (2008), Dale and Tanner (2012), and Sakellariou and Papadopoulos (2020) that CLIL fostered linguistic development. It was also found that the participants were more confident in terms of speaking English. This finding corroborated that of Christopher et al. (2012) that CLIL contributed to confidence regarding speaking in the target language. Hawamdeh and Soykan (2021) also underlined that international students of the English language need an inclusive learning environment to study a foreign language. CLIL can enable this kind of an environment, and as Fazio and Isidori (2021) noted recently, technological tools provide effective aid and allow to devote more time to language learning. According to them, in accordance with the findings of our study, CLIL classes and language learning objectives are believed to be blended with PE-specific vocabulary and objectives to communicate subject knowledge (Fazio and Isidori, 2021).

### Limitations

One of the limitations of this study is that the literature on the topic of this study indicates less research. Although there are a number of CLIL studies in primary and secondary school physical education classes, there is a lack of CLIL studies in amateur or professional sports teams. In addition, there is a scarcity of CLIL studies conducted with disabled individuals. Due to this scarcity, the discussion of the findings is carried out by referring to those conducted in physical education classes. Another limitation is that this study is designed as a small-scale study. For this reason, the sample size is not large enough to make generalizations. Other limitation is that due to the descriptive nature of the study, this study does not attempt to assess the linguistic development of the participants as it is beyond the scope. Further research can employ a pre-test-post-tense measurement of linguistic development in the target language.

## Conclusion

Disabled individuals experience more difficulties than healthy people in their lives. This study evaluated the effects of the CLIL program on motivation, morale, and wellbeing by integrating English instruction into the training of wheelchair basketball players. The results indicated that CLIL benefited the disabled individuals in terms of self-image, motivation, social skills, and speaking skills in the target language. CLIL has been frequently used worldwide as an effective method of teaching languages by incorporating foreign languages into a number of contents. As students are required to learn both subject area knowledge and foreign languages simultaneously, CLIL makes way for a more natural way to learn a foreign language. The application of CLIL to contents like physical education and sports is scarce, which calls for a need for further research.

## Data availability statement

The datasets presented in this study can be found in online repositories. The names of the repository/repositories and accession number(s) can be found in the article/supplementary material.

## Ethics statement

The studies involving human participants were reviewed and approved by the Ethical Committee Board of Near East University. The patients/participants provided their written informed consent to participate in this study.

## Author contributions

All authors listed have made a substantial, direct, and intellectual contribution to the work, and approved it for publication.
